# Is Chronic Low Back Pain Associated with the Prevalence of Coronary Heart Disease when Genetic Susceptibility Is Considered? A Co-Twin Control Study of Spanish Twins

**DOI:** 10.1371/journal.pone.0155194

**Published:** 2016-05-12

**Authors:** Matt Fernandez, Juan R. Ordoñana, Jan Hartvigsen, Manuela L. Ferreira, Kathryn M. Refshauge, Juan F. Sánchez-Romera, Marina B. Pinheiro, Stephen J. Simpson, John L. Hopper, Paulo H. Ferreira

**Affiliations:** 1 Arthritis & Musculoskeletal Research Group, Faculty of Health Sciences, The University of Sydney, Sydney, Australia; 2 Murcia Twin Registry, Department of Human Anatomy and Psychobiology, University of Murcia and IMIB-Arrixaca, Murcia, Spain; 3 Department of Sports Science and Clinical Biomechanics, University of Southern Denmark, Odense M, Denmark; 4 Nordic Institute of Chiropractic and Clinical Biomechanics, University of Southern Denmark, Odense M, Denmark; 5 Musculoskeletal Division, The George Institute for Global Health, Sydney Medical School, The University of Sydney, Sydney, Australia; 6 Institute of Bone and Joint Research, The Kolling Institute, Sydney Medical School, The University of Sydney, NSW, Australia; 7 Charles Perkins Centre, School of Biological Sciences, The University of Sydney, Sydney, Australia; 8 Australian Twin Registry, Centre for Molecular, Environmental, Genetic, and Analytic Epidemiology, The University of Melbourne, Victoria, Australia; Universidad Europea de Madrid, SPAIN

## Abstract

**Objective:**

To investigate the chronic low back pain and coronary heart disease relationship, after adjusting for relevant confounders, including genetics.

**Methods:**

In a cross-sectional design, 2148 twins were recruited from the Murcia Twin Registry, Spain. The exposure was chronic LBP and the outcomes were myocardial infarction and other coronary heart diseases—lifetime and in the last 2 years–based on standardized health-related questionnaires. First, logistic regression analysis investigated associations of the total sample followed by a matched co-twin control analyses, with all complete twin pairs discordant for chronic LBP utilised, separated for zygosity—dizygotic (DZ) and monozygotic (MZ) pairs, which adjusted for shared familial factors, including genetics.

**Results:**

Chronic LBP pain is associated with lifetime myocardial infarction [odds ratio (OR) = 2.69, 95% confidence interval (CI) = 1.35–5.36], other coronary heart diseases over a lifetime (OR = 2.58, 95% CI: 1.69–3.93) and in the last two years (OR = 2.19, 95% CI: 1.33–3.60), while there was a borderline association with myocardial infarction in the last 2 years (OR = 2.64, 95% CI: 0.98–7.12). Although the magnitude of the association remained or increased in the co-twin control analyses, none reached statistical significance.

**Conclusion:**

Chronic LBP is associated with a higher prevalence of myocardial infarction and coronary heart disease. It is possible that this association remains even when controlling for genetics and early shared environment, although this should be investigated with larger samples of twins discordant for LBP.

## Introduction

The 2013 global burden of disease study identified low back pain (LBP) as the greatest contributor to disability worldwide [1]. The lifetime, annual, and point prevalence rates of LBP have been reported as 38.9%, 38.0%, and 18.3% respectively [2]. Most cases of LBP appear to follow a chronic-episodic course, significantly impacting the health care system, individuals, and families [3]. In the United States, health care costs amongst those with back pain have steadily increased, with the total indirect and direct costs estimated to be greater than $100 billion annually [4].

A number of health related co-morbidities have been found to be associated with LBP [5], including cardiovascular disease. Regarded as the number one cause of death worldwide [6], cardiovascular disease is responsible for almost 4.1 million deaths per year in Europe [7]. Approximately 1.8 million of these deaths have been attributed to coronary heart disease (also known as ischemic heart disease), which is the most common cause of cardiovascular disease. In the United States cardiovascular disease has an annual health care cost of approximately $475 billion [8], representing 17% of overall national health expenditure. Whilst a steady decline in the mortality rates has been reported [9], the burden remains high with one in every six deaths attributed to coronary heart disease in the United States in 2009 [10].

The evidence regarding the association between LBP and coronary heart disease is conflictive, with a number of studies showing a relationship with back pain severity [11] and mortality rate due to coronary heart disease [12, 13], while others show no association between these specific disease entities [14, 15]. Recent studies have found that the prevalence of coronary heart disease is highest among individuals with spinal pain (concurrent LBP and neck pain) as opposed to LBP alone [16, 17]. Furthermore, chronic musculoskeletal [18–20] and wide spread pain have also been associated with coronary heart disease [21–23].

An alternate way of unravelling the conflictive relationship between LBP and coronary heart disease is to investigate the influence of genetics and the early shared environment on the relationship between these diseases. This is in light of the overall heritability contribution to LBP, estimated to be as high as 67% [24], and heritability estimates for death from coronary heart disease have been estimated as 57% for males and 38% for females [25]. The implementation of a co-twin control study design, where one twin has the condition while the other co-twin does not, allows for controlling and eliminating the effects of genetic and early environmental influences on the LBP and co-morbidities [26, 27], such as coronary heart disease. The purpose of the present cross-sectional study is to investigate whether chronic LBP is associated with the prevalence of coronary heart disease, including myocardial infarction after adjusting for genetic and early environmental influences, using a co-twin control design.

## Materials and Methods

### Study sample and data collection

The study sample of this cross-sectional study originated from the Murcia Twin Registry (MTR) in Spain and comprised of identical monozygotic (MZ) and non-identical dizygotic (DZ) twins born in multiple births between 1940 and 1966. A complete description of recruitment methods and waves of data collection implemented by the MTR are provided elsewhere [28]. Briefly, baseline data collection on health-related (self-reported) information for the current study took place via standardized questionnaires between 2009 and 2011, for opposite sex, male-male, and female-female twin pairs—followed for 2, 3 and 4 years respectively. Data collection took place by way of telephone or face-to-face interviews.

### Ethics statement

MTR participants were contacted through postal letter invitation, which contained information regarding the objectives of the registry and study information. Twins were later contacted by telephone, with oral informed consent obtained prior to any data collection taking place. Similarly, written informed consent was obtained when participants were involved in face-to-face interviews. All MTR procedures, including informed consent and data collection for this study, was approved by the Murcia University Ethical Committee. National regulations regarding personal data protection were followed, as were applicable institutional and governmental regulations concerning the ethical use of human volunteers.

### Zygosity assessment

In 338 twin pairs, zygosity identification was carried out by DNA testing utilising a Short Tandem Repeats (STR) approach based on 14 autosomal loci plus amelogenin gender determination. When this was not possible, a 12-item questionnaire, focusing on the degree of resemblance and mistaken identity was used. This questionnaire has an agreement with DNA testing in approximately 96% of cases corresponding with zygosity [28].

### Assessment of exposure: LBP

The main exposure investigated in this study was participants’ reports of chronic LBP. Participants who answered ‘yes’ to the following question: “have you ever suffered from chronic LBP” were considered cases. This question originated from the Spanish National Health Survey [29], with chronic LBP defined and explained to participants as the presence of pain in the lower back area that lasted at least six months or longer (including recurrent episodes).

### Assessment of outcomes: coronary heart related diseases

The outcome of interest was symptoms of coronary heart related diseases, where participants were asked the following questions: (i) “have you ever suffered a myocardial infarction?” (ii) “have you ever suffered from other coronary heart diseases?” (iii) “have you suffered a myocardial infarction in the last 2 years?” and (iv)“have you suffered from other coronary heart disease in the last 2 years?” Participants who answered ‘yes’ confirmed that a medical doctor had diagnosed myocardial infarction or coronary heart disease and were then considered as cases for the specific outcome.

### Assessment of potential confounders

Given their known association with LBP and coronary heart disease, we investigated smoking status, engagement in physical activity and body mass index (BMI) as potential baseline confounders. Participants were asked about smoking habits, with answers dichotomised as ex/never smoker or current/occasional smoker. Physical activity was examined by participants’ engagement in leisure physical activity and was based on four options: i) “I do not practice exercise. My leisure time is mostly sedentary (reading, watching TV, movies etc)”; ii) “Some sport or physical activity occasionally (walking, gardening, soft gym, light efforts etc)”; iii) “Regular physical activity several times a month (tennis, jogging, swimming, cycling, team sports etc)”; iv) “Physical training several times a week”. Responses to the physical activity variable were dichotomised into sedentary/no physical activity engagement in recreational physical activity or mild/moderate/vigorous physical activity engagement. BMI was calculated by dividing the individuals’ body weight in kilograms by the square of their height in meters and was used as a continuous variable. Smoking status and leisure physical activity questions originated from the Spanish National Health Survey questionnaire [29].

Descriptive analyses were conducted for all included variables. The exposure variable was chronic LBP and the outcomes included “myocardial infarction at anytime”, “myocardial infarction in the last 2 years”, “other coronary heart diseases” and “other coronary heart diseases in the last 2 years”.

The data was analysed in two stages: (i) total sample analysis; and (ii.a) co-twin control analysis for DZ twins followed by (ii.b) co-twin control analysis for MZ twins. Multivariate regression models were adjusted by smoking, physical activity, BMI, as well as age and gender where relevant (e.g. age and gender were omitted from the co-twin control analysis for MZ twins). Estimated associations were presented as odds ratios (OR) with 95% confidence intervals (CI). Statistical significance was set at p<0.05 for all multivariate models, with STATA 12 statistical software (StataCorp LP, College Station, TX, version 12.0) used for data analysis. We interpreted results based on the magnitude and plausibility of the associations, as well as the confidence intervals, rather than exclusively on p-values.

### Total sample analysis

All participants were included in the total sample cross-sectional analyses, using complete or incomplete twin pairs regardless of twin concordance status. In these analyses, genetics and early shared environmental factors are not accounted for. Unconditional multivariate logistic regression models were used based on the sandwich or Huber-White variance estimator, which adjusts estimated standard errors to account for data dependence between twins in a pair and provides statistical tests that are robust to model assumptions.

### Within-pair case-control analysis

To control for the influence of genetic and early shared environmental factors, separate co-twin control analyses were conducted for DZ and MZ pairs. A co-twin control analysis was performed using conditional logistic regression, with only complete and discordant twin pairs e.g. one twin reported chronic LBP but not the co-twin. Variables adjusted for in the total sample were retained and used for the co-twin control analysis, excluding irrelevant variables (e.g. age and gender in MZ models). This procedure allows for a comparison of models across all analytical stages (from total sample to MZ twins). These sequential analytical steps gradually eliminate genetic and familial confounding, allowing for a more direct estimate of the relationship between variables. In theory, when the association between two variables increases (or remains) in sequence, from the total sample analysis (no adjustment for genetics or early shared environment) to a DZ co-twin control analysis (adjustment for early shared environment and approximately 50% of genetics), and then to a MZ co-twin control analysis (adjustment for early shared environment and approximately 100% of genetics), the relationship between the two variables is more direct and possibly consistent with a causal link or path. Alternatively, if the association between the two variables decreases, then confounding (total or partial) may be present, influencing the initially observed relationship.

## Results

### Characteristics of the sample

A total of 2,148 participants formed the study sample for the cross-sectional analysis (Tables 1 and 2). Briefly, the mean age of all participants was 53.7 (SD = 7.3) years, with male participants making up 45% of the study sample. Participants who suffered a myocardial infarction or other coronary heart diseases (lifetime and in the in the last 2 years) were more likely to be older, suffer chronic LBP, male (only for myocardial infarction—lifetime and last 2 years), and heavier (BMI).

**Table 1:** Baseline characteristics of participants for ‘myocardial infarction’ lifetime and in the last two years.

Variables	Yes MI lifetime		No MI lifetime		Yes MI last 2 years		No MI last 2 years		Total	
	n	Mean ± SD or %	n	Mean ± SD or %	n	Mean ± SD or %	n	Mean ± SD or %	n	Mean ± SD or %
**Age (years)**	38	58.4±7.7	2111	53.6±7.3	17	57.1±7.6	2131	53.6±7.3	2148	53.7±7.3
**Gender–Male**	28	73.7%	945	44.8%	9	52.9%	964	45.2%	973	45.3%
**Gender—Female**	10	26.3%	1166	55.2%	8	47.1%	1167	54.8%	1176	54.7%
**MZ Male**	7	18.4%	281	13.3%	3	17.7%	285	13.4%	288	13.4%
**MZ Female**	5	13.2%	412	19.5%	4	23.5%	412	19.3%	417	19.4%
**DZ Male**	8	21.1%	354	16.8%	4	23.5%	358	16.8%	362	16.9%
**DZ Female**	3	7.9%	404	19.1%	3	17.7%	404	19.0%	407	19.0%
**DZ opposite**	15	39.5%	660	31.3%	3	17.7%	672	31.5%	675	31.4%
Chronic LBP*	17	44.7%	677	32.1%	9	52.9%	685	32.2%	694	32.3%
**Smoker**^¥^	15	39.5%	880	41.9%	6	35.3%	888	41.9%	895	41.8%
**Physical activity**^£^	21	55.3%	1144	54.4%	9	52.9%	1156	54.4%	1165	54.4%
**BMI**^	37	28.8±4.0	2055	27.4±4.5	17	29.6±4.4	2074	27.4±4.5	2092	27.4±4.5

*indicate chronic low back pain of at least 6 months duration;

^¥^ indicate current smokers;

^£^indicate engagement in leisure physical activity;

^ indicate body mass index;

*SD* = standard deviation; *LBP* = low back pain; *MZ* = monozygotic; *DZ* = dizygotic

**Table 2:** Baseline characteristics of participants for ‘other coronary heart diseases’ lifetime and in the last two years.

Variables	Yes CHD lifetime		No CHD lifetime		Yes CHD last 2 years		No CHD last 2 years		Total	
	n	Mean ± SD or %	n	Mean ± SD or %	n	Mean ± SD or %	n	Mean ± SD or %	n	Mean ± SD or %
**Age (years)**	115	58.9±7.2	2032	53.4±7.2	79	58.8±7.4	2067	53.5±7.3	2147	53.7±7.3
**Gender–Male**	57	49.6%	916	45.1%	36	45.6%	936	45.3%	973	45.3%
**Gender—Female**	58	50.4%	1116	54.9%	43	54.4%	1131	54.7%	1174	54.7%
**MZ Male**	10	8.7%	278	13.7%	7	8.9%	281	13.6%	288	13.4%
**MZ Female**	18	15.7%	398	19.6%	14	17.7%	402	19.5%	416	19.4%
**DZ Male**	25	21.7%	337	16.6%	15	19.0%	346	16.7%	362	16.9%
**DZ Female**	16	13.9%	390	19.1%	14	17.7%	392	19.0%	406	18.9%
**DZ opposite**	46	40.0%	629	31.0%	29	36.7%	646	31.3%	675	31.4%
Chronic LBP*	59	48.7%	638	31.4%	38	48.1%	656	31.8%	694	32.4%
**Smoker**^¥^	32	27.8%	861	42.6%	19	24.1%	874	42.5%	893	41.8%
**Physical activity**^£^	64	55.7%	1100	54.3%	42	53.2%	1121	54.4%	1164	54.4%
**BMI**^	110	28.4±4.9	1980	27.4±4.5	76	28.7±5.2	2013	27.4±4.5	2090	27.4±4.5

⁺indicates other coronary heart diseases diagnosed by doctor including: arrhythmia, pacemaker, angina, mitral stenosis, auricular fibrillation, pericarditis, coronary insufficiency, tachycardia, coronary valve disease, murmur, bypass, rheumatic myocarditis, cardiac arrest during labour, prolapse mitral valve, right bundle branch block and acute coronary syndrome;

*indicate chronic low back pain of at least 6 months duration;

^¥^ indicate current smokers;

^£^indicate engagement in leisure physical activity;

^ indicate body mass index;

*SD* = standard deviation; *LBP* = low back pain; *MZ* = monozygotic; *DZ* = dizygotic

### Chronic LBP and myocardial infarction

**Fig 1:**
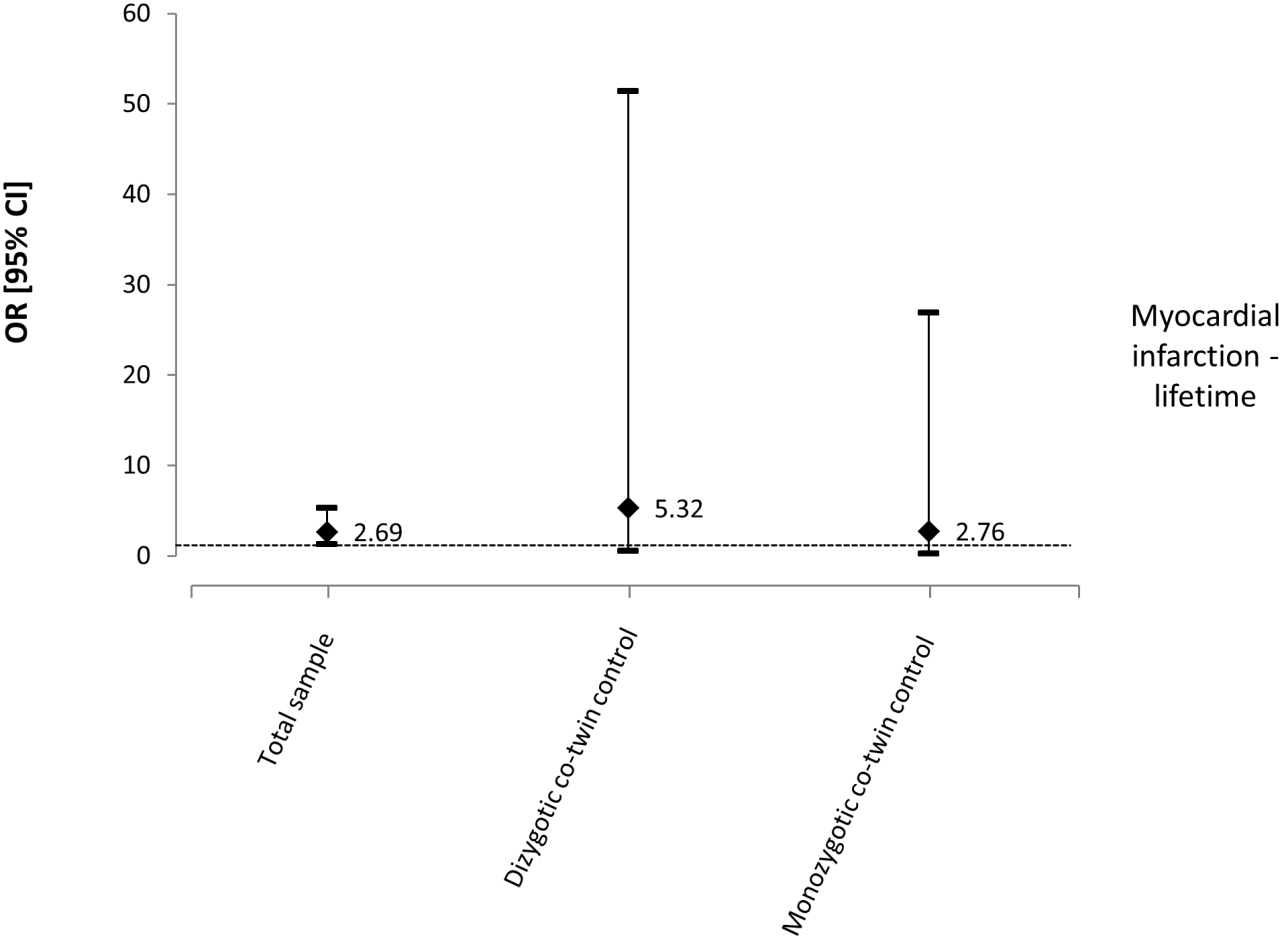
Odds ratios and 95% CIs from multivariate models for the relationship between chronic LBP and myocardial infarction—lifetime.

For the outcome of ‘lifetime myocardial infarction’, the variables age, gender, smoking, leisure physical activity and BMI were entered into the multivariate model for the total sample analysis. Chronic LBP was associated with a higher prevalence of myocardial infarction for the total sample (n = 2075, OR: 2.69, 95% CI: 1.35 to 5.33, p = 0.005) (Table 3). For the co-twin control analysis, a total of 344 pairs of twins were discordant for chronic LBP, including 230 DZ and 114 MZ pairs. Gender (except for MZ twins), smoking, leisure physical activity and BMI were considered confounding variables and entered into the multivariate analysis. An increase in the magnitude of the association was observed when the analyses were separated for DZ (OR: 5.32, 95% CI: 0.55 to 51.48, p = 0.149) and MZ twins (OR: 2.76, 95% CI: 0.28 to 26.96, p = 0.382), although none of these were statistically significant (Fig 1).

**Table 3:** Total sample analysis and co-twin control analyses for myocardial infarction lifetime and in the last 2 years.

**Myocardial infarction—lifetime**	**OR (95% CI)**	**P-value**
**Total sample analysis (n = 2075)**		
Chronic LBP	2.69 (1.35–5.33)^a^	**0.005**
**Co-twin control–DZ only (n = 230 pairs)**		
Chronic LBP	5.32 (0.55–51.48)^b^	0.149
**Co-twin control–MZ only (n = 114 pairs)**		
Chronic LBP	2.76 (0.28–26.96)^c^	0.382
**Myocardial infarction—last 2 years**	**OR (95% CI)**	**P-value**
**Total sample analysis (n = 2074)**		
Chronic LBP	2.64 (0.98–7.12)^a^	0.055
**Co-twin control–DZ only (n = 230 pairs)**		
Chronic LBP	3.34 (0.27–40.68)^b^	0.345
**Co-twin control–MZ only (n = 114 pairs)**		
Chronic LBP	1.83 (0.16–20.49)^c^	0.623

^a^Adjusted for: age, gender, smoking, leisure physical activity and body mass index

^b^Adjusted for: gender, smoking, leisure physical activity and body mass index

^c^Adjusted for: smoking, leisure physical activity and body mass index

### Chronic LBP and myocardial infarction in the last two years

**Fig 2:**
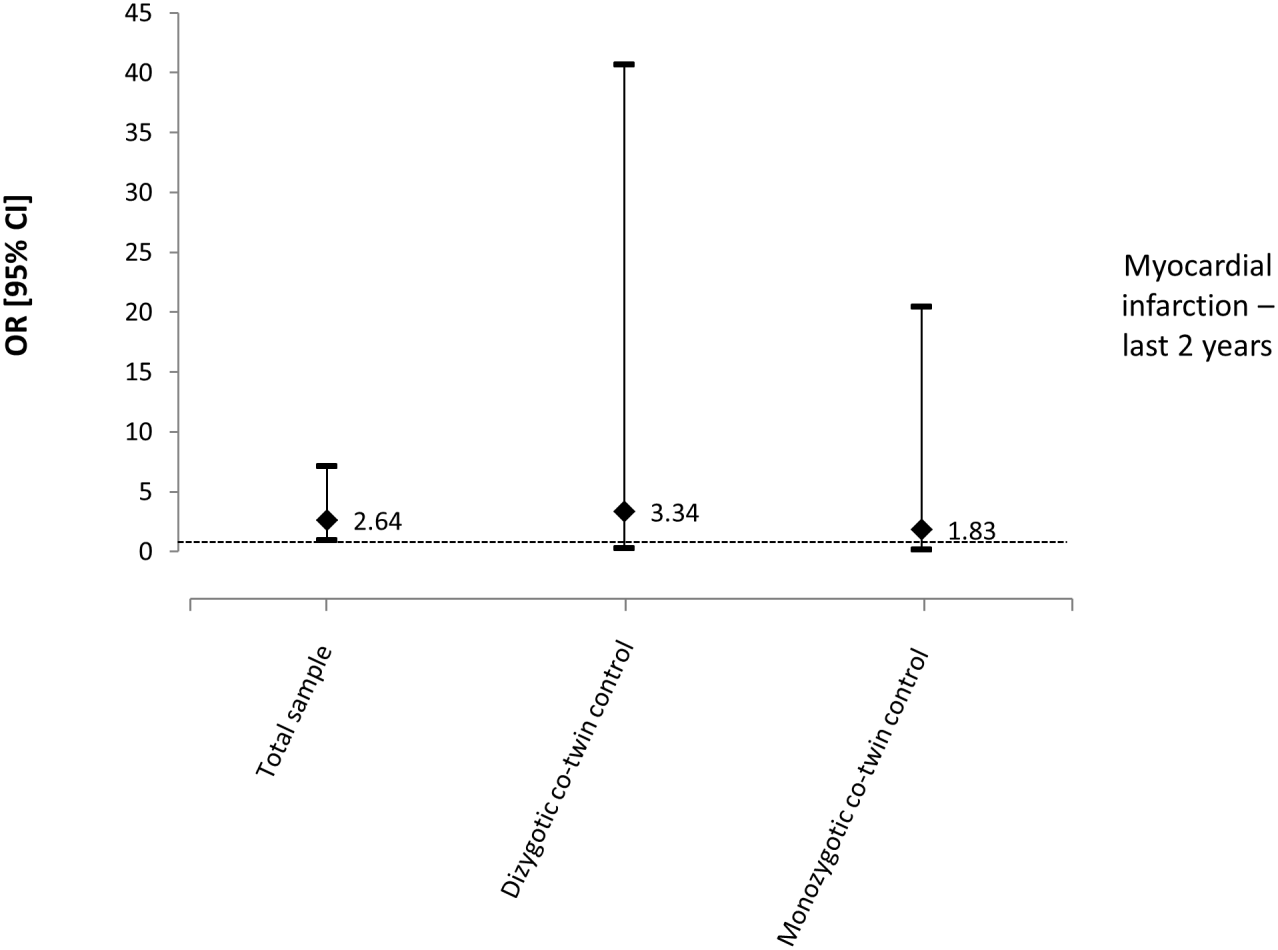
Odds ratios and 95% CIs from multivariate models for the relationship between chronic LBP and myocardial infarction—last 2 years.

For the outcome of ‘myocardial infarction in the last two years’, the variables age, gender, smoking, leisure physical activity, and BMI were entered into the multivariate model for the total sample analysis. There is a borderline association between chronic LBP and suffering/experiencing a myocardial infarction in the last two years for the total sample (n = 2074), although it did not reach statistical significance (OR: 2.64, 95% CI: 0.98 to 7.12, p = 0.055) (Table 3). For the co-twin control analysis, a total of 344 pairs of twins were discordant for chronic LBP, with 230 pairs being DZ and 114 MZ. Gender (except for MZ twins), smoking, leisure physical activity and BMI were considered confounding variables and entered into the multivariate analysis. An increase (not statistically significant) in the association with chronic LBP was observed for DZ (OR: 3.34, 95% CI: 0.27 to 40.68, p = 0.345) but not for MZ twins (OR: 1.83, 95% CI: 0.16 to 20.49, p = 0.623) (Fig 2).

### Chronic LBP and other coronary heart diseases

**Fig 3:**
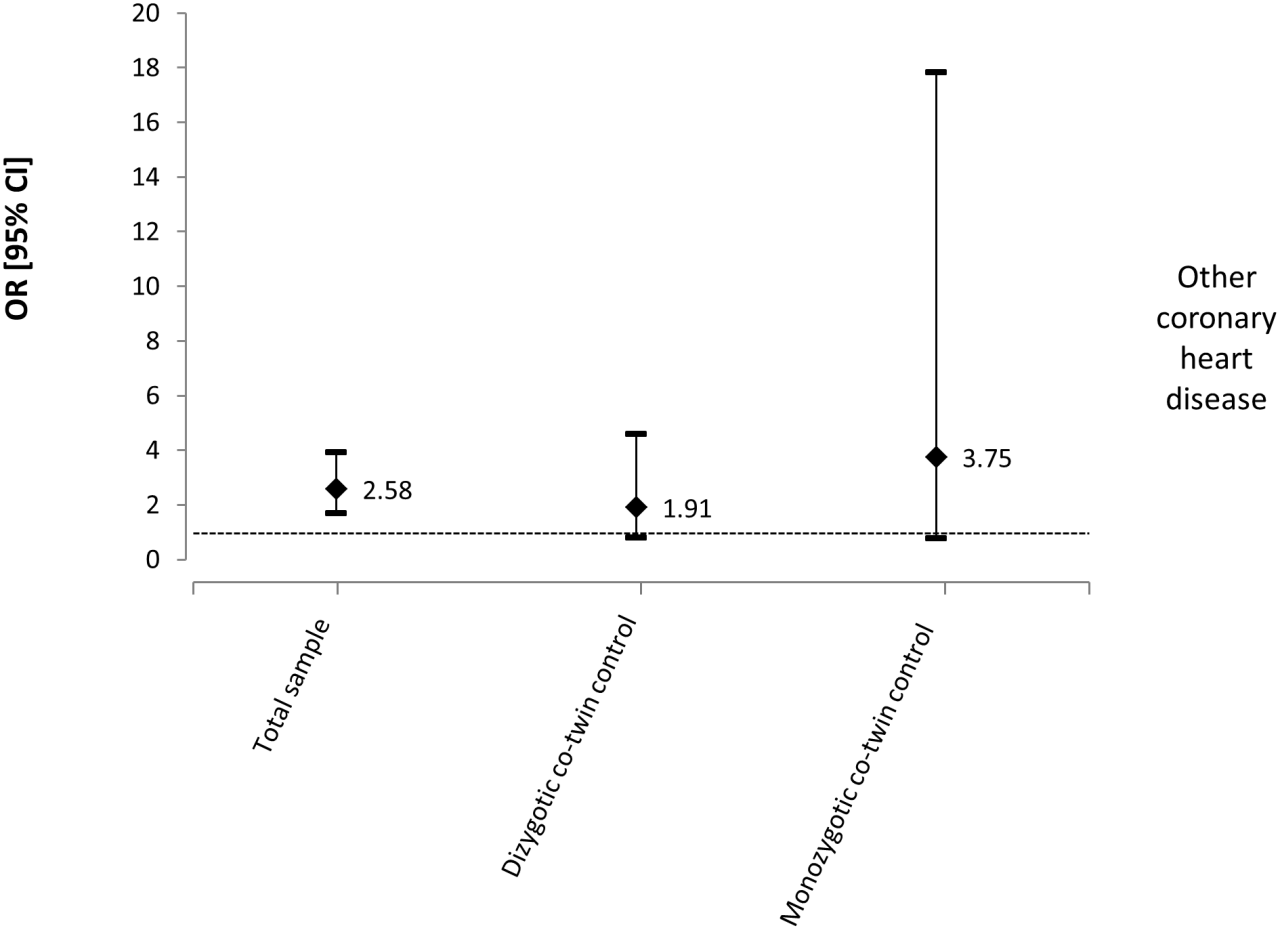
Odds ratios and 95% CIs from multivariate models for the relationship between chronic LBP and other coronary heart diseases—lifetime.

For the outcome of ‘other lifetime coronary heart diseases’, the variables age, gender, smoking, leisure physical activity and BMI were entered into the multivariate model for the total sample analysis. Chronic LBP was associated with a higher prevalence of other lifetime coronary heart diseases for the total sample (n = 2073, OR = 2.58, 95% CI: 1.69 to 3.93, p<0.001) (Table 4). For the co-twin control analysis, a total of 344 pairs of twins were discordant for chronic LBP, with 230 pairs being DZ and 114 MZ. Gender (except for MZ twins), smoking, leisure physical activity and BMI were considered confounding variables and entered into the multivariate analysis. An increase in the magnitude of the association was observed for MZ twins, although none of these analyses reached statistical significance (DZ: OR: 1.91, 95% CI: 0.80 to 4.61, p = 0.147, MZ: OR: 3.75, 95% CI: 0.79 to 17.83, p = 0.097) (Fig 3).

**Table 4:** Total sample analysis and co-twin control analyses for other coronary heart diseases lifetime and in the last 2 years.

**Other coronary heart diseases—lifetime**	**OR (95% CI)**	**P-value**
**Total sample analysis (n = 2073)**		
Chronic LBP	2.58 (1.69–3.93)^a^	**<0.001**
**Co-twin control–DZ only (n = 230 pairs)**		
Chronic LBP	1.91 (0.80–4.61)^b^	0.147
**Co-twin control–MZ only (n = 114 pairs)**		
Chronic LBP	3.75 (0.79–17.83)^c^	0.097
**Other coronary heart diseases—last 2 years**	**OR (95% CI)**	**P-value**
**Total sample analysis (n = 2073)**		
Chronic LBP	2.19 (1.33–3.60)^a^	**0.002**
**Co-twin control–DZ only (n = 230 pairs)**		
Chronic LBP	1.48 (0.59–3.69)^b^	0.402
**Co-twin control–MZ only (n = 114 pairs)**		
Chronic LBP	2.75 (0.54–13.85)^c^	0.219

^a^Adjusted for: age, gender, smoking, leisure physical activity and body mass index

^b^Adjusted for: gender, smoking, leisure physical activity and body mass index

^c^Adjusted for: smoking, leisure physical activity and body mass index

### Chronic LBP and other coronary heart diseases in the last two years

**Fig 4:**
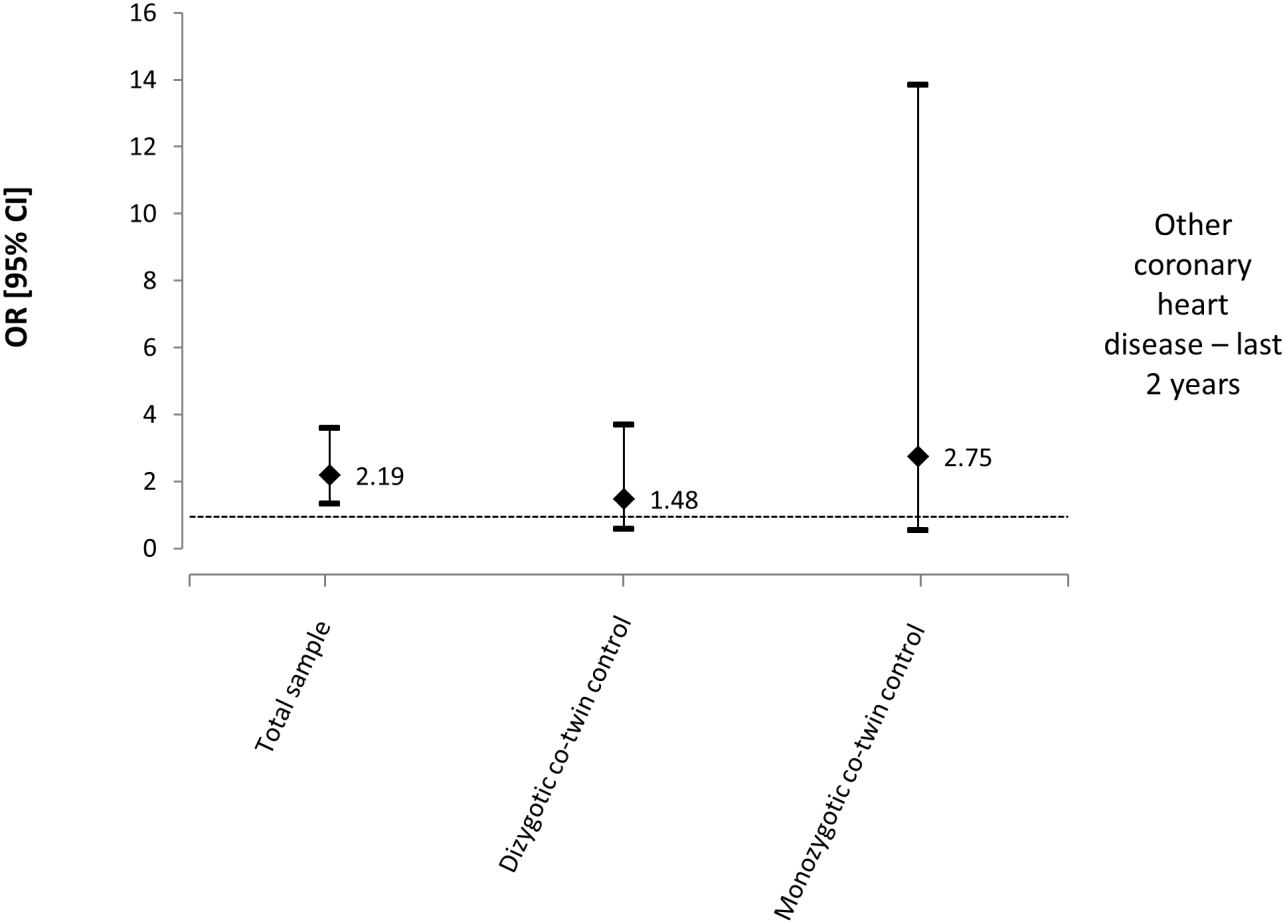
Odds ratios and 95% CIs from multivariate models for the relationship between chronic LBP and other coronary heart diseases—last 2 years.

For the outcome of ‘other coronary heart diseases in the last 2 years’, the variables age, gender, smoking, leisure physical activity, and BMI were entered into the multivariate model for the total sample analysis. Chronic LBP was associated with a higher prevalence of other heart diseases in the last two years for the total sample (n = 2073, OR: 2.19, 95% CI: 1.33 to 3.60, p = 0.002) (Table 4). For the co-twin control analysis, a total of 344 pairs of twins were discordant for chronic LBP, with 230 pairs being DZ and 114 MZ. Gender (except for MZ twins), smoking, leisure physical activity and BMI were considered confounding variables and entered into the multivariate analysis. The magnitude of the association increased for MZ twins, although none of these analyses reached statistical significance (DZ: OR: 1.48, 95% CI: 0.59 to 3.69, p = 0.402, MZ: OR: 2.75, 95% CI: 0.54 to 13.85, p = 0.219) (Fig 4).

## Discussion

The results from our cross-sectional analyses showed an association between chronic LBP and lifetime myocardial infarction and a borderline association for myocardial infarction in the last two years. Similarly, there was a relationship between chronic LBP and other coronary heart diseases, both at lifetime and in the last two years, when twins were considered part of the total sample. Further analytical steps, which partially or fully controls for genetics and early shared environment, showed an overall pattern of increase in the magnitude of the association between LBP and coronary heart related diseases, although no statistically significant relationship was observed.

The magnitude of the OR for MZ twins (were highest levels of adjustment are achieved) in the prevalence analysis suggests that although this association is no longer significant, we should not assume that genetics and the early shared environment are confounding the association between chronic LBP, myocardial infarction and other coronary heart diseases. Although the cross-sectional nature of our study limits the possibility of identifying causal relationships, the co-twin control design provides some insight, suggesting the possibility that the association is not completely due to genetics and familial factors and that the loss of significance could reflect a decrease of power due to the reduction in sample size when using discordant pairs and the low prevalence of the outcome. This interpretation is based on the magnitude of the OR in the MZ analysis and plausibility of the effects of LBP on other coronary heart diseases (including myocardial infarction), rather than a narrowed inspection of p-values, which is known to be of limited clinical interpretation and problematic [30].

There are a number of (non-twin) cross-sectional studies that have investigated the relationship between LBP and coronary heart disease. Overall our results are in general agreement, including a study which found an association between chronic spinal pain and the prevalence of coronary heart disease in adults 18 years or older (OR: 1.3, 95%CI: 1.0 to 1.7) [18]. The presence of severe back pain was significantly associated with the prevalence of cardiovascular disease and myocardial infarction in elderly women (OR: 3.0, 95% CI: 2.0 to 4.6) [11]. General chronic musculoskeletal pain is also associated with higher prevalence estimates of cardiovascular disease in people over 65 years (OR: 1.8, 95%CI: 1.45 to 2.30) [19], and myocardial infarction (OR: 2.47, 95%CI: 1.43 to 4.28) [20].

Differences in our study compared to previous studies might be due to variations in study design, such as the definition of our exposure, study samples and outcome measures employed. For example, our study focused on chronic LBP of at least 6 months duration whereas others measured chronic back pain in the last 12 months [11, 18] or chronic musculoskeletal pain either on the day of the survey [19] or in the last month [20]. A feature of our study is the implementation of a co-twin control design, which allowed the evaluation of genetic and shared familial confounding. Whilst our results might have been affected by power and need to be interpreted with a degree of caution, this method to our knowledge has not been implemented in previous cross-sectional studies of chronic LBP and coronary heart related diseases, therefore providing new insights in this field. Future research should consider high-quality longitudinal designs to explore causal paths, along with larger sample sizes, for example, combining twin data from different datasets in order to evaluate a larger number of discordant twins on a global scale. The International Network of Twin Registries may be a potential source in this regard, as it promotes international collaboration for twin research [31] and its utilisation may provide more precise estimates and solid conclusions on familial confounding, including genetics across societies.

Given the prevalence of coronary heart disease related to chronic LBP, it is important to consider a number of potential mechanisms underlying this possible relationship. Physiologically, an unhealthy lifestyle adopted due to pain can have a considerable impact on a number of co-morbidities, including coronary heart disease. This seems plausible, given the level of disability and subsequent inactivity associated with LBP [32]. Furthermore, general ‘stress’, elevated cortisol levels [33], sympathetic-parasympathetic imbalances [34] and the presence of central sensitization (i.e., pro-inflammatory cytokines) [35] has been found to be associated with cardiovascular disease and musculoskeletal pain [36]. Psychologically, pain can affect mental health status, triggering symptoms of depression and/or anxiety [37]. Whether the physiological and/or psychological dysfunctions that accompany chronic LBP are cause or effect of this condition, cardiovascular diseases are also associated with these mechanisms [23]. Another potential mechanism which may partially explain this relationship is the presence of atherosclerosis (i.e., atheromatous lesions of lumbar arteries) in the abdominal aorta in people with low back pain [38]. Atherosclerosis limits blood supply and nutritional exchange to the lumbar intervertebral discs, promoting the risk of disc degeneration [39]. Moreover, a high BMI accelerates aortic stiffness, and may act as a preceding lesion prior to aortic plaque formation [40]. Clinicians should therefore be encouraged to routinely monitor individuals with a history of LBP and support them in adopting healthier lifestyle choices [23].

A major strength of our study, in particular, was a discordant co-twin control analysis on a population-based sample of twins. However, whilst discordant MZ twin pairs allow genetics and the early shared environment to be controlled for, there is the possibility of bias through 'non-measured' or ‘non-shared’ variables that differ between members of a MZ pair, which may act as residual confounders, for example occupation exposure. Despite our sample data being obtained via rigorous interviews, the quality of self-reported chronic LBP, myocardial infarction and other coronary heart diseases was based on health-related questionnaires, which could be interpreted differently by different responders, and this may be seen as a limitation, despite the moderate to good agreement between self-report and medical record data for myocardial infarction [41]. There may have also been participants with chronic LBP excluded as they were not diagnosed with coronary heart-related disease by a medical doctor but may have subclinical coronary heart disease (i.e., the presence of some but not all criteria to achieve a diagnosis), such as a family history of cardiovascular disease [42].

The screening questionnaire provided no information on the severity or duration of chronic LBP, only addressing a single time point, hence it is not known whether people with a history of chronic LBP either recovered, had intermittent or continuous symptoms. In addition, participants may have experienced an episode of LBP during their lifetime and may be subjected to ‘recall bias’, particularly if the pain experience occurred some time ago. However, there is a recurrent, fluctuating course associated with LBP [43] i.e., acute-on-chronic episodes, which frequently affects a high percentage of individuals in the working population over a long period [44]. Furthermore, additional analysis showed that the prevalence of chronic LBP in our study was similar to the prevalence of LBP reported in the last 2 years—in the same cohort (e.g. lifetime myocardial infarction: 44.7% vs. 42.1%, lifetime coronary heart disease: 48.7% vs. 43.9%). More comprehensive measures in the future may assist, for example, not only for LBP but also in the identification of risk factors for the future onset (or transition to) chronic widespread pain among chronic LBP patients [45]. Prevalence of coronary heart disease may also be due to residual confounding, with chronic pain patients frequently experiencing unmeasured confounders such as symptoms of anxiety or depression [46]–the latter strongly associated with cardiovascular disease [47]. Moreover, our study did not account for hypertension, lipid levels and medication use such as non-steroidal anti-inflammatory drugs, which may increase risk of cardiovascular disease [48].

## Conclusion

In summary, chronic LBP is associated with an increased prevalence of lifetime myocardial infarction and other coronary heart diseases. The observed relationship could be independent of familial confounding, including genetics, although this needs to be confirmed in further studies.

## References

[1] Vos T, Barber RM, Bell B, Bertozzi-Villa A, Biryukov S, Bolliger I, et al Global, regional, and national incidence, prevalence, and years lived with disability for 301 acute and chronic diseases and injuries in 188 countries, 1990–2013: a systematic analysis for the Global Burden of Disease Study 2013. The Lancet. 2015;386(9995):743–800. 10.1016/S0140-6736(15)60692-4 PMC4561509 26063472

[2] Hoy D, Bain C, Williams G, March L, Brooks P, Blyth F, et al A systematic review of the global prevalence of low back pain. Arthritis Rheum. 2012;64(6):2028–37. 10.1002/art.34347 22231424

[3] Hoy D, Brooks P, Blyth F, Buchbinder R. The Epidemiology of low back pain. Best Practice & Research Clinical Rheumatology. 2010;24(6):769–81. 10.1016/j.berh.2010.10.002 21665125

[4] Martin BI, Deyo RA, Mirza SK, et al EXpenditures and health status among adults with back and neck problems. JAMA. 2008;299(6):656–64. 10.1001/jama.299.6.656 18270354

[5] Hestbaek L, Leboeuf-Yde C, Manniche C. Is low back pain part of a general health pattern or is it a separate and distinctive entity? A critical literature review of comorbidity with low back pain. J Manipulative Physiol Ther. 2003;26(4):243–52. . 12750659 10.1016/s0161-4754(03)00003-4

[6] Tarnoki AD, Tarnoki DL, Molnar AA. Past, present and future of cardiovascular twin studies. Cor Vasa. 2014;56(6):e486–e93. 10.1016/j.crvasa.2014.07.005

[7] Nichols M, Townsend N, Scarborough P, Rayner M. Cardiovascular disease in Europe: epidemiological update2013 2013-10-14 00:00:00. 3028–34 p. 10.1093/eurheartj/eht356 24014390

[8] Heidenreich PA, Trogdon JG, Khavjou OA, Butler J, Dracup K, Ezekowitz MD, et al Forecasting the Future of Cardiovascular Disease in the United States: A Policy Statement From the American Heart Association. Circulation. 2011;123(8):933–44. 10.1161/CIR.0b013e31820a55f5 21262990

[9] Ford ES, Greenlund KJ, Hong Y. Ideal Cardiovascular Health and Mortality From All Causes and Diseases of the Circulatory System Among Adults in the United States. Circulation. 2012;125(8):987–95. 10.1161/circulationaha.111.049122 22291126 PMC4556343

[10] Go AS, Mozaffarian D, Roger VL, Benjamin EJ, Berry JD, Borden WB, et al Executive Summary: Heart Disease and Stroke Statistics—2013 Update: A Report From the American Heart Association. Circulation. 2013;127(1):143–52. 10.1161/CIR.0b013e318282ab8f 23283859

[11] Vogt MT, Nevitt MC, Cauley JA. Back problems and atherosclerosis. The Study of Osteoporotic Fractures. Spine. 1997;22(23):2741–7. . 9431608 10.1097/00007632-199712010-00008

[12] Penttinen J. Back pain and risk of fatal ischaemic heart disease: 13 year follow up of Finnish farmers. BMJ. 1994;309(6964):1267–8. 7888848 10.1136/bmj.309.6964.1267 PMC2541802

[13] Zhu K, Devine A, Dick IM, Prince RL. Association of Back Pain Frequency With Mortality, Coronary Heart Events, Mobility, and Quality of Life in Elderly Women. Spine. 2007;32(18):2012–8 10.1097/BRS.0b013e318133fb82 17700450

[14] Penttinen J. Risk of myocardial infarction among subjects visiting a doctor because of back disorder. A case—control study in Finnish farmers. Spine. 1995;20(24):2774–6. . 8747258 10.1097/00007632-199512150-00021

[15] Heliovaara M, Makela M, Aromaa A, Impivaara O, Knekt P, Reunanen A. Low back pain and subsequent cardiovascular mortality. Spine. 1995;20(19):2109–11. . 8588167 10.1097/00007632-199510000-00008

[16] Strine TWM, Hootman JMPATCF. US National Prevalence and Correlates of low Back and Neck Pain Among Adults. Arthritis & Rheumatism Arthritis Care & Research. 2007;57(4):656–65. 17471542 10.1002/art.22684

[17] Fernandez-de-Las-Penas C, Alonso-Blanco C, Hernandez-Barrera V, Palacios-Cena D, Jimenez-Garcia R, Carrasco-Garrido P. Has the prevalence of neck pain and low back pain changed over the last 5 years? A population-based national study in Spain. Spine Journal: Official Journal of the North American Spine Society. 2013;13(9):1069–76. 10.1016/j.spinee.2013.02.064 . 23578987

[18] Von Korff M, Crane P, Lane M, Miglioretti DL, Simon G, Saunders K, et al Chronic spinal pain and physical–mental comorbidity in the United States: results from the national comorbidity survey replication. Pain. 2005;113(3):331–9. 10.1016/j.pain.2004.11.010 15661441

[19] Ryan CG, McDonough S, Kirwan JP, Leveille S, Martin DJ. An investigation of association between chronic musculoskeletal pain and cardiovascular disease in the Health Survey for England (2008). Eur J Pain. 2014;18(5):740–50. 10.1002/j.1532-2149.2013.00405.x . 24167109

[20] Parsons S, McBeth J, Macfarlane GJ, Hannaford PC, Symmons DPM. Self-reported pain severity is associated with a history of coronary heart disease. European Journal of Pain. 2015;19(2):167–75. 10.1002/ejp.533 24890750 PMC4322478

[21] Lindgren H, Bergman S. Chronic musculoskeletal pain predicted hospitalisation due to serious medical conditions in a 10 year follow up study. BMC Musculoskel Disord. 2010;11(1):1–9. 10.1186/1471-2474-11-127 PMC2903507 20565826

[22] McBeth J, Symmons DP, Silman AJ, Allison T, Webb R, Brammah T, et al Musculoskeletal pain is associated with a long-term increased risk of cancer and cardiovascular-related mortality. Rheumatology (Oxford, England). 2009;48(1):74–7. 10.1093/rheumatology/ken424 . 19056799 PMC2639482

[23] Andersson HI. Increased mortality among individuals with chronic widespread pain relates to lifestyle factors: A prospective population-based study. Disabil Rehabil. 2009;31(24):1980–7. 10.3109/09638280902874154 . 19874076

[24] Ferreira PH, Beckenkamp P, Maher CG, Hopper JL, Ferreira ML. Nature or nurture in low back pain? Results of a systematic review of studies based on twin samples. European Journal of Pain. 2013;17(7):957–71. 10.1002/j.1532-2149.2012.00277.x 23335362

[25] Zdravkovic S, Wienke A, Pedersen NL, Marenberg ME, Yashin AI, De Faire U. Heritability of death from coronary heart disease: a 36-year follow-up of 20 966 Swedish twins. J Intern Med. 2002;252(3):247–54. 10.1046/j.1365-2796.2002.01029.x 12270005

[26] Pinheiro MB, Ferreira ML, Refshauge K, Colodro-Conde L, Carrillo E, Hopper JL, et al Genetics and the environment affect the relationship between depression and low back pain: a co-twin control study of Spanish twins. Pain. 2015;156(3):496–503. 10.1097/01.j.pain.0000460330.56256.25 00006396-201503000-00018. 25679471

[27] Dario A, Ferreira M, Refshauge K, Sánchez-Romera J, Luque-Suarez A, Hopper J, et al Are obesity and body fat distribution associated with low back pain in women? A population-based study of 1128 Spanish twins. Eur Spine J. 2015:1–8. 10.1007/s00586-015-4055-2 26084786

[28] Ordoñana JR, Rebollo-Mesa I, Carrillo E, Colodro-Conde L, García-Palomo FJ, González-Javier F, et al The Murcia Twin Registry: A Population-Based Registry of Adult Multiples in Spain. Twin Research and Human Genetics. 2013;16(Special Issue 01):302–6. 10.1017/thg.2012.66 23046559

[29] Igualdad MdSSSe, editor Encuesta Nacional de Salud 2011–2012 [Spanish National Health Survey 2011–2012]2012.: Ministerio de Sanidad Servicios Sociales e Igualdad.

[30] Nuzzo R. Scientific method: statistical errors. Nature. 2014;506(7487):150–2. 10.1038/506150a . 24522584

[31] Buchwald D, Kaprio J, Hopper JL, Sung J, Goldberg J, Fortier I, et al International Network of Twin Registries (INTR): Building a Platform for International Collaboration. Twin Research and Human Genetics. 2014;17(06):574–7. 10.1017/thg.2014.67 25431288

[32] Gore M, Sadosky A, Stacey BR, Tai K-S, Leslie D. The Burden of Chronic Low Back Pain: Clinical Comorbidities, Treatment Patterns, and Health Care Costs in Usual Care Settings. Spine. 2012;37(11):E668–E77. 10.1097/BRS.0b013e318241e5de 00007632-201205150-00022. 22146287

[33] Van Uum SHM, Sauvé B, Fraser LA, Morley-Forster P, Paul TL, Koren G. Elevated content of cortisol in hair of patients with severe chronic pain: a novel biomarker for stress. Stress (Amsterdam, Netherlands). 2008;11(6):483–8. 10.1080/10253890801887388 . 18609301

[34] Brotman DJ, Golden SH, Wittstein IS. The cardiovascular toll of stress. The Lancet. 370(9592):1089–100. 10.1016/S0140-6736(07)61305-1 17822755

[35] Giesecke T, Gracely RH, Grant MAB, Nachemson A, Petzke F, Williams DA, et al Evidence of augmented central pain processing in idiopathic chronic low back pain. Arthritis Rheum. 2004;50(2):613–23. 10.1002/art.20063 . 14872506

[36] Wang H, Schiltenwolf M, Buchner M. The Role of TNF-α in Patients With Chronic Low Back Pain—A Prospective Comparative Longitudinal Study. The Clinical Journal of Pain. 2008;24(3):273–8. 10.1097/AJP.0b013e31816111d3 00002508-200803000-00014. 18287835

[37] Linton SJ, Bergbom S. Understanding the link between depression and pain. Scandinavian Journal of Pain. 2011;2(2):47–54. 10.1016/j.sjpain.2011.01.005 29913734

[38] Kauppila LI. Atherosclerosis and Disc Degeneration/Low-Back Pain–A Systematic Review. Eur J Vasc Endovasc Surg. 2009;37(6):661–70. 10.1016/j.ejvs.2009.02.006 19328027

[39] Kurunlahti M, Tervonen O, Vanharanta H, Ilkko E, Suramo I. Association of atherosclerosis with low back pain and the degree of disc degeneration. Spine. 1999;24(20):2080–4. . 10543002 10.1097/00007632-199910150-00003

[40] Brunner EJ, Shipley MJ, Ahmadi-Abhari S, Tabak AG, McEniery CM, Wilkinson IB, et al Adiposity, obesity, and arterial aging: longitudinal study of aortic stiffness in the Whitehall II cohort. Hypertension. 2015;66(2):294–300. 10.1161/hypertensionaha.115.05494 . 26056335 PMC4490910

[41] Okura Y, Urban LH, Mahoney DW, Jacobsen SJ, Rodeheffer RJ. Agreement between self-report questionnaires and medical record data was substantial for diabetes, hypertension, myocardial infarction and stroke but not for heart failure. J Clin Epidemiol. 2004;57(10):1096–103. 10.1016/j.jclinepi.2004.04.005 . 15528061

[42] Pandey AK, Pandey S, Blaha MJ, Agatston A, Feldman T, Ozner M, et al Family history of coronary heart disease and markers of subclinical cardiovascular disease: where do we stand? Atherosclerosis. 2013;228(2):285–94. 10.1016/j.atherosclerosis.2013.02.016 . 23578356

[43] Croft PR, Macfarlane GJ, Papageorgiou AC, Thomas E, Silman AJ. Outcome of low back pain in general practice: a prospective study. BMJ (Clinical research ed). 1998;316(7141):1356–9. 10.1136/bmj.316.7141.1356 . 9563990 PMC28536

[44] Andersson GB. Epidemiological features of chronic low-back pain. Lancet (London, England). 1999;354(9178):581–5. 10.1016/s0140-6736(99)01312-4 . 10470716

[45] Viniol A, Jegan N, Brugger M, Leonhardt C, Barth J, Baum E, et al Even worse: Risk factors and protective factors for transition from chronic localized low back pain to chronic widespread pain in general practice—A cohort study. Spine. 2015 10.1097/brs.0000000000000980 . 25955187

[46] Dersh J, Polatin PB, Gatchel RJ. Chronic Pain and Psychopathology: Research Findings and Theoretical Considerations. Psychosom Med. 2002;64(5):773–86 00006842-200209000-00010. 12271108 10.1097/01.psy.0000024232.11538.54

[47] Rugulies R. Depression as a predictor for coronary heart disease: a review and meta-analysis1. Am J Prev Med. 2002;23(1):51–61. 10.1016/S0749-3797(02)00439-7 12093424

[48] Goodson NJ, Smith BH, Hocking LJ, McGilchrist MM, Dominiczak AF, Morris A, et al Cardiovascular risk factors associated with the metabolic syndrome are more prevalent in people reporting chronic pain: Results from a cross-sectional general population study. Pain. 2013;154(9):1595–602. 10.1016/j.pain.2013.04.043 23707277

